# Steroid-induced psychosis in context a SARS-CoV-2 pandemic. about a case

**DOI:** 10.1192/j.eurpsy.2021.834

**Published:** 2021-08-13

**Authors:** C. Martín Villarroel, L. Carpio Garcia, G. Belmonte García, J. Dominguez Cutanda, M. Sánchez Revuelta, J. Matsuura, M. Fernández-Torija Daza, E. García

**Affiliations:** Psiquiatría, Complejo Hospitalario Universitario de Toledo, Toledo, Spain

**Keywords:** SARS-CoV-2, corticosteroids, Psychiatric symptoms, steroid-induced psychosis

## Abstract

**Introduction:**

SARS-CoV-2 is having an important direct impact, and also due to treatments used such as corticosteroids. Among its effects, we have focused on psychosis.

**Objectives:**

The objective of this paper is to study, from following case, incidence of steroid-induced psychosis in context of COVID-19.

**Methods:**

A bibliographic search was performed from different database (Pubmed, TripDatabase) about psychiatric symptoms associated with use of corticosteroids during pandemic. 64-year-old woman with no psychiatric history, who is hospitalized for pneumonia secondary to SARS-Cov2 and treated with antibiotics, bronchodilators, and corticosteroids. At 4 days she began with injury and nihilistic delusions. The corticosteroids were progressively reduced, adding 2.5 mg Risperidone, resolving after ten days.

**Results:**

Corticosteroids are currently being used to treat the systemic inflammatory response associated with COVID-19, but they can produce other effects such as psychiatric symptoms (3-6%): 75% affective (mainly hypomanic symptoms); and 25% psychotic. Steroid-induced psychosis are characterized by confusion, delusions, and hallucinations, and they usually begin 3-4 days after onset, and resolve within a week. They are associated especially with oral systemic steroids and high doses: 1.3% with 40mg of prednisone, and 18% with 80mg; increased this incidence due to the greater use that is being made to treat COVID-19 and the higher doses used in severe cases (up to 120 mg).

**Conclusions:**

To conclude, we need to know characteristics of these episodes in order to be able to prevent and treat them properly (minimum effective dose and less time), since they will probably occur more frequently at this time.

